# The delay of motherhood: Reasons, determinants, time used to achieve pregnancy, and maternal anxiety level

**DOI:** 10.1371/journal.pone.0227063

**Published:** 2019-12-30

**Authors:** Leticia Molina-García, Manuel Hidalgo-Ruiz, Eva María Cocera-Ruíz, Esther Conde-Puertas, Miguel Delgado-Rodríguez, Juan Miguel Martínez-Galiano

**Affiliations:** 1 Department of Obstetrics and Gynecology, Complejo Hospitalario de Jaén, Andalucia, Jaén, Spain; 2 Department of Obstetrics and Gynecology, Hospital San Juan de la Cruz, Andalucia, Úbeda, Spain; 3 Department of Obstetrics and Gynecology, Hospital San Agustín, Andalucia, Linares, Spain; 4 Department of Health Sciences, University of Jaén, Andalucia, Jaén, Spain; 5 Consortium for Biomedical Research in Epidemiology and Public Health (CIBERESP), Comunidad de Madrid, Madrid, Spain; 6 Department of Nursing, University of Jaén, Andalucia, Jaén, Spain; China University of Science and Technology, CHINA

## Abstract

**Background:**

Fertility in recent decades in European countries such as Norway, Spain or United Kingdom has declined, while in others such as Portugal, it has remained relatively constant, and in others such as Germany fertility rated have risen. The determinants of this change in reproductive pattern can be explained by the cultural, social, and economic changes that took place in our society. Objective: to identify the principal reasons and independent determinants associated with the postponement of motherhood and document any association between the time taken to achieve successful pregnancy and maternal age, as well as the level of anxiety of these women.

**Methods:**

An observational study, including 326 women, was conducted in Spain with primiparous women, in which data was collected on sociodemographic, health, and pregnancy-related factors. Comparison of means (t-test or analysis of variance) and the analysis of covariance was used to estimate adjusted means for potential confounders.

**Results:**

Women in stable relationships became mothers at older ages (31.83±0.29) than those who were not (28.75±0.78) (p<0.001). Women who delayed motherhood for medical reasons had a mean age of 34.15 ± 0.88 years, compared to a mean of 30.52±0.36 years for personal reasons, and 27.51±1.39 years for other reasons. Mothers with an older age had a higher level of anxiety (p<0.05). The average time required to achieve pregnancy increased as maternal age increased, with an average time of 24 months for women with a mean age of 35.23±0.71 years compared to <3 months for women with a mean age of 29.44±0.39 years. Women ≥ 35 years were more likely to need medical assistance to achieve pregnancy (aOR = 12.07, 95% CI: 1.50–97.05; p = 0.019).

**Conclusions:**

Medical reasons were among those cited for delaying motherhood. The postponement of motherhood was associated with difficulty to achieve a successful pregnancy and a higher level of anxiety.

## Introduction

Fertility in recent decades in European countries such as Italy, Norway, Spain or United Kingdom has declined, while in others such as Portugal, it has remained relatively constant, and in others such as Germany or Lithuania fertility rated have risen [1]. The determinants of this change in reproductive pattern can be explained by the cultural, social, and economic changes that took place in our society, especially in the last third of the 20th century [2].

At present, there is no consensus for the appropriate age to achieve pregnancy. Some authors establish this age at 35 years [3–5], whereas others place it at 40 or even 44 years [2]. There is a definite increase in the number of women bearing children in the 30- and 40-year-old age groups. The total number of women who are 35 to 40 years in the United States is projected to increase 42%, and the percentage of births in this age group is projected to increase 37% [6]. Pregnancies in women with advanced age have increased in recent years. For example, in Spain in 2013, 27,875 births for women 40 years or older were recorded, which represented 6.7% of all births in Spain; and this percentage is increasing, with pregnancies in women 40 years and over accounting for 7.4% of all births in 2017. This situation is not unique to Spain, and pregnancy at an advanced age is also increasing in countries such as Japan, United Kingdom, Australia, and Chile, among others [7]. Pregnancy at an advanced maternal age is known to have consequences for maternal heathl and neonatal health [8,9].

One of the consequences that appears linked to late motherhood has to do with the biological limits of fertility such as the increase in infertility, miscarriage, and the use of assisted reproduction techniques [10–14].

The reasons that lead a woman to postpone the decision to be a mother have been explored by different studies [15–19]. Women plan to be mothers at a certain point in their life. For example, after obtaining work or training goals, and may delay the process over time in favor of economic, academic and professional stability [15–19]. In a 2018 National Fertility Survey of 14,446 women in Spain, it was found that 26.15% women over 35 who had not had children had delayed childbearing because they did not have a stable relationship, 3.25% because she was too young to be a mother, 13.27% had not wanted to be a mother, 3.08% had to continue studying, 17.94% for work reasons and reconciling family and work life, 10.56% for economic reasons, 17.57% for health reasons, and 8.18% for other reasons [15].

As mentioned above, other aspects of delayed maternity are the increase in time spent to successfully achieve pregnancy [13,20] and having to resort to assisted reproduction techniques [15,21–23]. In a population-based birth cohort study carried out with 4333 pregnant women in the urban area of Pelotas in Southern Brazil, mothers who used assisted reproductive technology were compared to mothers with spontaneous pregnancy; in general, women who required assisted reproductive technology were older than those with a spontaneous pregnancy [23].

Motherhood at an advanced age can lead to a higher level of anxiety during pregnancy and childbirth. Perinatal anxiety is highly prevalent and merits clinical attention. [24] Maternal anxiety has been associated with consequences for the health of the mother and the baby [25, 26]. The effect of maternal age on the level of anxiety is not clear in studies to date [26, 27].

The delay of motherhood has consequences for the mother and the newborn. The number of women who give birth at an older age is increasing. In order to confirm this trend, and thus be able to design adequate health policies aimed at prevention with the design of health programs in which these variables are taken into account in order to guarantee the best assistance to women, the following objectives were proposed: identify the main reasons reported by the women and independent determinants (objective factors) associated with the postponement of motherhood, as well as document whether there is an association between maternal age and the time taken to achieve successful pregnancy or the need for medical assistance, as well as the influence of maternal age on the level of anxiety.

## Materials and methods

An analytic observational study was conducted on pregnant women who gave birth during 2017 (from January to December) simultaneously in different hospitals in South Spain. The inclusion criteria were: primiparous pregnant women, with a singleton pregnancy, and who were not minors (>18 years). Women that had difficulty communicating in Spanish (language barrier) and women whose pregnancy was unplanned were excluded.

### Ethics approval and consent to participate

Ethical approval was obtained from the Ethics Committees of the hospitals participating in the study: Comité de Ética de la Investigación del Complejo Hospitalario Universitario de Granada (Committee of Ethics of Investigation of the Complejo Hospitalario Universitario de Granada), Comité de Ética de la Investigación del Hospital Reina Sofia de Córdoba (the Committee of Ethics of Investigation of the Reina Sofia Hospital), Comité de Ética de la Investigación del Complejo Hospitalario Universitario de Jaén; (the Committee of Ethics of Investigation of the Complejo Hospitalario Universitario de Jaén), Comité de Ética de la Investigación del Hospital de Úbeda (the Committee of Ethics of Investigation of the Ubeda Hospital) and Comité de Ética de la Investigación del Hospital de Linares (the Committee of Ethics of Investigation of the Linares Hospital). The informed consent was verbally obtained because no interventions were performed on the study. It was to answer a survey’s questions. It was more pragmatic to obtain a verbal consent. In order to obtain the verbal consent of the woman, first of all she was informed of the study, the objective of this. what was your participation (answer an interview), risks and the willingness of your participation. The woman raised the possible doubts that arose to solve them. If the woman verbally accepted it was included in the study. The different Ethics Committes authorized and were aware about verbally consent use on the study. Informed consent was obtained from the women, and the established protocols for the respective health centers were followed for access to medical records data and to conduct this type of study; with the objective to publish/disseminate the results to the scientific community.

The results of this study are part of a larger study. The principal outcome of the larger study was the appearance of pregnancy-associated pathology. The sample size was based on the study by Heras Pérez et al. in 2011 [2], in which the incidence of pregnancy-associated pathology in women older than 35 years was 29.2% compared with 15.8% in women 35 years or younger. To detect a difference between these figures (29.2% and 15.8%) with a power of 80% and a statistical significance of 5%, it was estimated that a sample size of 302 women would be needed. Considering an expected drop-out rate of 15%, a final total of 373 women were recruited. Women were selected consecutively. Only 13 women refused to participate.

### Data collection

Data were collected using a questionnaire which had previously been piloted. The questionnaire (closed and open items) was heteroadministered by qualified personnel (midwives) with knowledge of pregnancy, childbirth, and the puerperium, in the immediate postpartum period via an interview by the midwives who collaborated in the study. The majority of the data were obtained through the clinical interview conducted by a health professional following childbirth; the data were then completed with access to the clinical history and the pregnancy health document.

Data were collected on the sociodemographic variables of the pregnant women, as well as on variables related to obstetric antecedents, the current pregnancy, and main reason women delayed motherhood. To assess the level of maternal anxiety, the Spanish version of the Hospital Anxiety and Depression Scale (HADS), developed by Zigmond and Snaith (1983) was used [28]. Of the 14 items that make up the scale, 7 assess anxiety and the rest evaluate depression: Only those items that assess the level of anxiety were chosen and applied. This modified scale had already previously been used in women of the same sociodemographic characteristics as those in the study. [29]

### Data analysis

Continuous variables were assessed by comparison of means, t-test, or analysis of variance. The analysis of covariance was used to estimate adjusted means for potential confounders. For categorical variables, odds ratios (OR) and their 95% confidence intervals (95% CI) were calculated using logistic regression to adjust for confounding. Confounders were considered those variables which were non-intermediate variables and changed the coefficient of the main exposure (maternal age) by more than 10% in multivariate models. All analyses were performed using Stata 14 (College Station, TX).

## Results

A total of 326 women participated in the study. The mean age of participating women was 31.13 ± 5.37 years. Almost all, 99.1% (323), were Spanish, and 68.4% (223) were married. The mean income of 42.4% (122) was between 1000–1999 Euros per month. Women with university education made up 40.2% (131) of the women participating in the study and around 72.7% (237) worked during pregnancy. A previous history of miscarriage was reported in 24.5% (80), and 12.3% (40) had some type of pathology prior to pregnancy. 14.1% (46) required medical assistance to achieve pregnancy: 34.8% (16) required in vitro fertilization, 21.7% (10) artificial insemination, 17.4% (8) the prescription of some type of medication, 15.2% (7) intracytoplasmic sperm injection, 8.7% (4) oocyte donation, and 2.2% (1) sperm donation (Table 1).

**Table 1:** Characteristics of the study population.

Variable	
Mean age (SD)	31.13 (5.37)
Civil status, n (%)	
Single	71 (21.8)
Married	223 (68.4)
Defacto relationship	29 (8.9)
Divorced	3 (0.9)
Nationality	
Spanish, n (%)	323 (99.1)
Other	3 (0.9)
Education level, n (%)	
No education	6 (1.8)
Primary	22 (6.8)
Secondary	84 (25.7)
Upper secondary*	83 (25.5)
University	131 (40.2)
Income, n (%)	
< 1000 Euros	72 (25.0)
1000–1999 Euros	122 (42.4)
2000–2999 Euros	64 (22.2)
≥ 3000 Euros	30 (10.4)
Employed during pregnancy, n (%)	
No	89 (27.3)
Yes	237 (72.7)
Illness, n (%)	
No	286 (87.7)
Yes	40 (12.3)
Previous miscarriages, n (%)	
No	246 (75.5)
Yes	80 (24.5)
Attended antenatal education, n (%)	
No	132 (40.5)
Yes	194 (59.5)
Pregnancy follow-up, n (%)	
Public system	150 (46.0)
Private system	6 (1.8)
In both	170 (52.2)
Medical assistance to achieve pregnancy, n (%)	
No	280 (85.9)
Yes	46 (14.1)
Gestation at birth, mean (SD)	39.43 (1.41)

* Baccalaureate (equivalent of A levels)/Professional formation. Abbreviations: n, number; SD, standard deviation

Table 2 shows the association between maternal age and different sociodemographic variables. Women who had a partner became mothers at later ages (31.83 ± 0.29) than those who did not (28.75 ± 0.78) (p <0.001). In the same way, those with university studies were older (33.24 ± 0.38) compared to those with a lower level of education (p < 0.001). Unemployed women became mothers at a younger age (27.54 ± 0.58) than women who have any type of employment contract (p < 0.001). The increase in the level of monthly income was proportionally related to the increase in the maternal age at which the first child was born. Women with incomes lower than 1000 euros became mothers with a mean age of 28.06 ± 0.68 years, compared to 33.55 ± 0.79 years for those earning more than 3000 euros per month (p < 0.001).

**Table 2:** Association between maternal age and different sociodemographic variables.

Variable	Total n	Age m ± SEM	p-value
Marital status: Partner			< 0.001
No	74	28.75 ± 0.78	
Yes	252	31.83 ± 0.29	
Level of education			< 0.001
Primary	28	30.73 ± 1.44	
Secondary	167	29.56 ± 0.39	
University	131	33.24 ± 0.38	
Monthly household income			< 0.001
< 1000 Euros	72	28.06 ± 0.68	
1000–1999 Euros	122	31.49 ± 0.45	
2000–2999 Euros	64	32.22 ± 0.48	
≥ 3000 Euros	30	33.55 ± 0.79	
Employment type			< 0.001
Unemployed	84	27.54 ± 0.58	
Temporary contract	58	30.29 ± 0.61	
Indefinite contract	124	32.70 ± 0.40	
Own business/self-employed	38	33.08 ± 0.86	
Other	21	34.57 ± 0.94	

Abbreviations: m, mean; SEM, standard error of mean.

Table 3 shows the main reasons cited by women for delaying childbearing. In the crude analysis, differences were found between the reasons given for the delay of maternity; for example, mothers who argued medical reasons had an average age of 35.05 ± 0.77 while those who claimed personal reasons had an average age of 30.47 ± 0.38 (p<0.001). The changes observed in the multivariable analysis included women who delayed motherhood for medical reasons with a mean age of 34.15 ± 0.88 years compared to a mean of 30.52 ± 0.36 years for women who had delayed for personal reasons, or for other reasons 27.51 ± 1.39 years–the youngest group. The association between maternal age and the different reasons for delaying motherhood was significant among the group that cited medical reasons (health problems resulting in recommendation against getting pregnant and infertility problems; for example: diseases, usually chronic such as autoimmune diseases, diabetic women with poor metabolic control, among others; women with pathological processes without definitive diagnosis, etc.”) (p < 0.001) and other reasons (p = 0.036) with respect to those who cited personal reasons (reference group). In women who indicated employment (reconciling work and family life) or economic reasons, the association was not significant (p > 0.05). Other reasons included 46.7% (7) indicating that it was not the right time before, 33.3% (5) not knowing the right person to have children with, and 20% (3) considering themselves as being too young to be a mother.

**Table 3:** Reasons reported by women for delaying childbearing and motherhood.

Variable	Total, n	Crude Analysis		Multivariate Analysis	
		Age m, SEM	p-value	Age ma*, SEM	p-value
Reason for delaying motherhood			<0.001		
Personal	210	30.47 ± 0.38		30.52 ± 0.36	ref.
Employment (reconciling work and family life)	57	32.35 ± 0.51		31.63 ± 0.70	0.156
Economic	12	30.11 ± 1.81		31.56 ± 1.53	0.508
Medical problems	32	35.05 ± 0.77		34.15 ± 0.88	<0.001
Other	15	27.90 ± 1.25		27.51 ± 1.39	0.036

* Adjusted for education level, income level, maternal smoking habit, history of previous miscarriage, and presence of medical pathology prior to pregnancy. Abbreviations: CI, confidence interval; m, mean; SEM, standard error of mean.

Table 4 analyzes the influence of maternal age on the time taken to achieve pregnancy successfully. The average time required to achieve pregnancy was greater as maternal age increased; with an average time of 24 months for women with a mean age of 35.38 ± 0.71 years compared to <3 months for women with a mean age of 29.27 ± 0.37 years. This association remained significant (p < 0.001) when adjusting for maternal education level, income level, maternal smoking habit, previous history of miscarriage, and presence of pathology prior to pregnancy.

**Table 4:** Influence of maternal age on the time taken to successfully achieve pregnancy.

Variable	Crude Analysis						Multivariate Analysis					
	Age (years) m ± SEM					p-value	Age (years) ma* ± SEM					p-value
Duration (months)	<3	3–6	>6–12	>12–24	>24		<3	3–6	>6–12	>12–24	>24	
	29.27 ± 0.37	30.53 ± 0.65	32.32 ± 0.83	33.58 ± 0.99	35.38 ±0.71	< 0.001	29.44 ± 0.39	30.58 ± 0.72	31.77 ± 0.73	32.45 ± 0.90	35.23 ± 0.71	< 0.001

* Adjusted for education level, income level, maternal smoking habit, history of previous miscarriage, and presence of medical pathology prior to pregnancy. Abbreviations: m, mean; SEM, standard error of mean

Table 5 shows the relationship between the need for medical assistance to achieve pregnancy and maternal age. Age was stratified into 4 groups: <25 years, 25–29 years, 30–34 years and ≥ 35 years. A positive association was established between maternal age and the need for medical assistance to achieve pregnancy. Women ≥ 35 years were more likely to need medical assistance to achieve a successful pregnancy than those of younger ages (aOR = 12.07, 95% CI: 1.50–97.05; p = 0.019), with maternal age acting as a risk factor in this case.

**Table 5:** Association between maternal age and the need for medical assistance to achieve successful pregnancy, stratified by age.

Variable	Total n	Age (years)			
		< 25 n (%)	25–29 n (%)	30–34 n (%)	≥ 35 n (%)
Medical Assistance					
No	280	32 (96.97)	69 (97.18)	112 (89.60)	67 (69.07)
Yes	46	1 (3.03)	2 (2.82)	13 (10.40)	30 (30.93)
OR (95% CI)		1 ref.	0.93 (0.08–10.61)	3.71 (0.47–29.48)	14.33 (1.87–109.80)
p-value			0.952	0.214	0.010
aOR* (95% CI)		1 ref.	0.76 (0.07–8.85)	3.03 (0.37–24.77)	12.07 (1.50–97.05)
p-value			0.828	0.301	0.019

* Adjusted for education level, income level, maternal smoking habit, history of previous miscarriage, and presence of medical pathology prior to pregnancy. Abbreviations: CI, confidence interval; n, number.

A significant association was detected between the level of anxiety presented by the mother and age (adjusted for maternal education level, income level, maternal smoking habit, previous history of miscarriage, and presence of pathology prior to pregnancy). The women with high anxiety had a mean age of 32.58 ± 0.83 years compared with those with lower anxiety levels, 30.48 ± 0.37 years (p = 0.022). Anxiety was assessed regarding the presence of a condition before pregnancy: it occurred in 39.5% of women with a chronic condition versus 33.6% of healthy women before pregnancy (OR = 1.29, 95% CI = 0.67–2.48). This OR was not modified after adjusting for age, income level, maternal smoking, previous history of miscarriage, and conditions during pregnancy (aOR = 1.29, 95% CI = 0.66–2.55). Anxiety was present in 37.8% of women needing assisted reproductive technology -ART- vs. 32.7% in the remaining women with a planned pregnancy (OR = 1.25, 95% CI = 0.65–2.40). After adjusting for the same variables above mentioned the relationship was not significant (aOR = 1.00, 95% CI = 0.47–2.10).

## Discussion

Several objectives were proposed in this study, such as identifying the main reasons reported by the women for delaying maternity, identify independent determinants (objective factors) associated with the postponement of motherhood, as well as document whether there is an association between maternal age and the time taken to achieve successful pregnancy or the need for medical assistance, and evaluate the influence of maternal age on the level of anxiety. In our results, having a partner, university level education, a higher income level, and job stability (type of contract) was associated with motherhood at a later age. Women indicated medical reasons and other reasons, such as it not being the right time before, as reasons for delaying motherhood.

An association was identified between maternal age and the time taken to achieve pregnancy once she decided to try. Furthermore, women who became mothers at an age older than 35 were more likely to need medical assistance to get pregnant. Women who became mothers at an older age were found to have a higher level of anxiety than younger women.

Montilva, in a study carried out in South America, explored the reasons for delaying motherhood and identified the need for the development of a professional career for stability in the labor market and consolidation of academic training, as well as finding a stable relationship [17]; coinciding with the results of our study. Additionally, Alamillos Guardiola found that women face motherhood in a planned manner and delay the process in favor of economic and professional stability [16]; in line with our results. Youth unemployment, temporary contracts, and unstable labor market conditions generate a context of economic "insecurity" that leads to the postponement of the first birth, due to the impossibility of making long-term binding decisions [19, 30–32]. These findings are in line with our results, where an indefinite contract that gives stability is associated with motherhood at an older age. This may be because achieving this employment contract in the labor market takes time while the required work experience is acquired.

Among the reasons cited by women for delaying motherhood were medical reasons; and this was the most prominent reason in relation to an advanced maternal age. This finding coincides with other published research [11,12,33]. As opposed to other studies [15], for the women participating in our study reasons related to employment (combining family and work) were not a significant reason for delaying motherhood. Our results, in which women delay motherhood for other reasons—such as that it was not the right time or not knowing the right person to have a child with—coincide with the National Fertility Survey carried out in Spain [15].

The time taken to achieve a successful pregnancy increased linearly with the increase in maternal age. This finding is consistent with that of Schmidt et al, who also found differences between the time taken to achieve pregnancy and age; with older women taking more time [13]. López Garrido et al., in a retrospective descriptive study of 491 pregnant women in Spain who achieved a desired spontaneous pregnancy, determined the influential factors on the mean time required to achieve pregnancy and highlighted a significant influence of maternal age [34]. They estimated a mean time of 6.8 cycles in women 35 years or younger and 10.9 cycles in those over 35 years, in other words, the older the women the more time it will take to achieve a pregnancy; in line with other authors [35] and with our results. Fisher et al. found, in an Australian study, that older women had fewer pregnancies achieved spontaneously than those who were mothers at a younger age [36]; in line with our results and other studies [15,21–23,37].

The mean age of the women with the highest anxiety level was higher than the mean age of those who presented lower levels; however, this association was only significant for the highest anxiety level. Similar results were obtained by Velásquez [38], who found a relationship between the maternal age of primiparous women and their level of anxiety, with a higher level of anxiety in the older primiparous women. In contrast to our results, Van Heyningen et al., in a cross-sectional study conducted in South Africa on 376 women, did not identify maternal age as a predictor of the pregnant woman's anxiety level [39]. The mode of conception (spontaneous or with medical help) was not associated in our results with the level of postpartum anxiety in line with the results of Lardon et al. [40] This may be due to the fact that once pregnancy and childbirth have been achieved, the possible anxiety that can result in not achieving pregnancy and childbirth decreases when the woman has achieved the goal that was set: having a child [41]. Therefore, we believe that this variable has not influenced the level of postpartum anxiety in mothers.

### Strengths and weaknesses of the study

The present study has its strengths and weaknesses. One advantage of our sample is representative of a reference population. The questionnaire used to collect the information has been previously tested. It is unlikely that an information bias exists as the collected data and the way the developed questions were carried out did not require having a high level of education (understandable for all education levels) to could be understood by all the participants regardless of their level of education. In terms of weaknesses, we cannot discard a memory bias, but if present, we think it would be a non-differential bias because the type of information collected and the short time period at which the questionnaire was administered. A selection bias associated with non-responders is unlikely to have had an influence on the results, as the majority of women selected agreed to participate. Only 13 woman refused participation and nor was there any reason to believe that those who did not participate would have had performed differently to those who did.

## Conclusion

A higher level of education, a higher level of income, job stability via an employment contract, as well as living as a couple, are determinants associated with motherhood at older ages. In addition, medical reasons are the among the reasons most cited by women for delaying motherhood. The delay in the decision to be a mother was associated with the difficulty to achieve pregnancy due to age and with the need for some kind of medical assistance, such as assisted reproduction techniques, to achieve a successful pregnancy.

## References

[1] Legazpe Moraleja N. Decisiones de formación de la pareja y maternidad en las mujeres españolas. Revista de Economía Aplicada. 2016, 24(72), 23–45.

[2] Heras Pérez B.; Gobernado Tejedor J.; Mora Cepeda P.; Almaraz Gómez A. La edad materna como factor de riesgo obstétrico. Resultados perinatales en gestantes de edad avanzada. Prog. Obs. Ginecol. 2011, 54(11), 575–580.

[3] Monleón Sancho J.; Baixauli C.; Minguez Milio J.; García Román N.; Plana A.; Monleón J. El concepto de primípara añosa. Prog. Obs. Ginecol. 2002, 45(9), 384–390

[4] Stufflebeam D.; Shinkfield JA. Evaluación Sistemática, Guía Teórica y Práctica, 1^a^ ed; Paidós Ibérica, Madrid, 1987.

[5] Sociedad Española de Ginecología y Obstetricia. Recomendaciones sobre la asistencia al parto. 2007. http://www.perinatalandalucia.es/file.php?file=%2F20%2F29.Recomendaciones_SEGO_asistencia_parto_normal.pdf (Accessed: 1 Junio, 2018).

[6] Gindoff P.R.; Jewelewicz R. Reproductive potential in the older woman. Fertil Steril. 1986, 46(6), 989–1001 10.1016/s0015-0282(16)49869-9 3536609

[7] Martínez Galiano J.M. La maternidad en madres de 40 años. Rev cub. salud pública. 2016, 42 (3), 451–458.

[8] Molina García L.; Hidalgo Ruiz M.; Arredondo López B.; Colomino Ceprián S.; Delgado Rodríguez M.; Martínez Galiano J.M. Maternal Age and Pregnancy, Childbirth and the Puerperium: Obstetric Results. J. Clin. Med. 2019, 8, 672. 10.3390/jcm8050672 PMC6571680 31086046

[9] Molina-García L, Hidalgo-Ruiz M, Cámara-Jurado AM, Fernández-Valero MJ, Delgado-Rodríguez M, Martínez-Galiano JM. Newborn Health Indicators Associated with Maternal Age during First Pregnancy. International Journal of Environmental Research and Public Health. 2019; 16(18):3448. 10.3390/ijerph16183448 PMC6765882 31533243

[10] Barros Delgadillo C.J.; Castañeda Tenorio M.; Aguayo González P.; Muñoz Manrique C. Consecuencias perinatales de embarazos logrados mediante reproducción asistida versus embarazos espontáneos. Ginecol. Obstet. Mex. 2018, 86(11), 732–731.

[11] Donoso E.; Carvajal J.A.; Vera C.; Poblete J.A. La edad de la mujer como factor de riesgo de mortalidad materna, fetal, neonatal e infantil. Rev. méd. Chile. 2014, 142, 168–174. 10.4067/S0034-98872014000200004 24953104

[12] Fernández O.; Masoli I. Fertilidad después de los 40 años. Rev. méd. Clín. Las Condes. 2009, 20(1), 16–21.

[13] Schmidt L.; Sobotka T.; Bentzen J.G.; Nyboe A. Demographic and medical consequences of the postponement of parenthood. Hum Reprod Update. 2012, 18(1), 29–43 10.1093/humupd/dmr040 21989171

[14] Balasch J.; Gratacós E.; Delayed childbearing: effects on fertility and the outcome of pregnancy. Curr Opin Obstet Gynecol. 2012, 24(3), 187–93. 10.1097/GCO.0b013e3283517908 22450043

[15] Instituto Nacional de Estadística. Demografía y población: Encuesta de Fecundidad. 2018 https://www.ine.es/dyngs/INEbase/es/operacion.htm?c=Estadistica_C&cid=1254736177006&menu=ultiDatos&idp=1254735573002 (Accessed: 1 Marzo, 2019).

[16] Alamillos Guardiola M.C. La maternidad tardía: expresión contemporánea del patriarcado occidental. Rev. Antropol. Exp. 2016, 16: 213–221. Disponible en línea: 10.17561/rae.v0i16.2241 (se accede en el 12 de Abril de 2019).

[17] Montilva M. Postergación de la maternidad de mujeres profesionales jóvenes en dos metrópolis latinoamericanas. Utopìa y Praxis Latinoamericana. 2008, 13(41), 69–79.

[18] Nunes Lopes M.; Lovato Dellazzana-Zanon L.; Gonçalves Boeckel M. A multiplicidade de papéis da mulher contemporânea e a maternidade tardia. Temas psicol. 2014, 22(4), 917–928.

[19] Ní Bhrolcháin M.; Beaujouan E. Fertility postponement is largely due to rising educational enrolment. Popul Stud. 2012, 66 (3), 311–327. 10.1080/00324728.2012.697569 PMC3479627 22889178

[20] Correia S.; Rodrigues T.; Barros H. Socioeconomic variations in female fertility impairment: a study in a cohort of Portuguese mothers. BMJ Open. 2014, 4(1). 10.1136/bmjopen-2013-003985 PMC3902378 24384900

[21] Manzur A.; Macaya R.; Gajardo G. Inseminación intrauterina en mayores de 38 años, ¿vale la pena?. Rev. peru. ginecol. obstet. 2012, 58 (1), 11–16.

[22] Fitzpatrick K.E.; Tuffnell D.; Kurinczuk J.J.; Knight M. Pregnancy at very advanced maternal age: a UK population-based cohort study. BJOG. 2017, 124(7), 1097–1106. 10.1111/1471-0528.14269 27581343 PMC5484369

[23] Ginar da Silva S.; Dâmaso Bertoldi A.; Freitas da Silveira M.; Rodrigues Domingues M.; Evenson K.R.; Silva dos Santos I. Assisted reproductive technology: prevalence and associated factors in Southern Brazil. Rev Saúde Pública. 2019, 53, 13 10.11606/S1518-8787.2019053000737 30726494 PMC6390642

[24] Dennis CL, Falah-Hassani K, Shiri R. Prevalence of antenatal and postnatal anxiety: systematic review and meta-analysis. Br J Psychiatry. 2017 5;210(5):315–323. 10.1192/bjp.bp.116.187179 28302701

[25] Dunkel-Schetter C. Psychological science on pregnancy: stress processes, biopsychosocial models, and emerging research issues. Annu Rev Psychol 2011;62:531–558 10.1146/annurev.psych.031809.130727 21126184

[26] Romero-Gutiérrez G, Rocha-Morales D, Ruiz-Treviño AS. Resultados de la aplicación de la escala de Hamilton modificada en el diagnóstico de ansiedad materna durante el puerperio inmediato. Ginecol Obstet Mex 2013;81:180–185 23720929

[27] Giardinelli L, Innocenti A, Benni L, Stefanini MC, Lino G, Lunardi C, et al Depression and anxiety in perinatal period: prevalence and risk factors in an Italian sample. Arch Womens Ment Health. 2012 2;15(1):21–30. 10.1007/s00737-011-0249-8 22205237

[28] Vázquez Valverde C.; Jiménez Franco F. Depression and mania In Clinical Measurement in Psychiatry and Psychology, 1^a^ ed; Bulbuena Vilarrasa A., Berrios G.E., Fernández de Larrinoa Palacios P., eds.; Barcelona, Spain, 2003.

[29] Martinez-Galiano JM, Delgado-Rodriguez M. La educación maternal como recurso para reducir la ansiedad que genera la proximidad del proceso de parto, Revista argentina de clinica psicológica, 2014, 23, (3), 261–266.

[30] Beets G. The Demography of the Age at First Birth: The Close Relationship between Having Children and Postponement En The Future of Motherhood in Western Societies. Beets G., Schippers J., te Velde E. eds.; New York, 2010.

[31] Mills M.; Rindfuss R.; McDonald P.; te Velde E. Why do people postpone parenthood? Reasons and social policy incentives. Hum Reprod Update. 2011, 17(6), 848–860. 10.1093/humupd/dmr026 21652599 PMC3529638

[32] Sobotka T. Shifting parenthood to advanced reproductive ages: Trends, causes and consequences En A Young Generation Under Pressure? Financial situation and ‘rush hour of life’ of the cohorts 1970–1985 in a generational comparison, 1^a^ ed; BerlinHeidelberg, Springer-Verlag; 2010, pp. 129–154.

[33] Esteve, A.; Devolder, D.; Domingo, A. La infecundidad en España: tic-tac, tic-tac, tic-tac!!!. Bellaterra Centre d'Estudis Demogràfics. 2016.

[34] López Garrido B.; Gil Pita R.; García Alfaro M.; Varela Quintela L.; García Ruiz N. Tiempo medio en alcanzar un embarazo deseado y factores influyentes. Matronas profesión. 2013, 14 (3–4), 74–81.

[35] Moreno Rosset C.; Antequera Jurado R.; Jenaro Río C.; Gómez Sánchez Y. La Psicología de la Reproducción: la necesidad del psicólogo en las Unidades de Reproducción Humana. Clínica y Salud. 2009, 20(1), 79–90.

[36] Fisher J.; Wynter K.; Hammarberg K.; McBain J.; Gibson F.; Boivin J.; et al Age, mode of conception, health service use and pregnancy health: a prospective cohort study of Australian women. BMC Pregnancy Childbirth. 2013, 13, 88 10.1186/1471-2393-13-88 23565589 PMC3622566

[37] Barros Delgadillo C.J.; Castañeda Tenorio M.; Aguayo González P.; Muñoz Manrique C. Consecuencias perinatales de embarazos logrados mediante reproducción asistida versus embarazos espontáneos. Ginecol Obstet Méx. 2018, 86(11), 732–731

[38] Velásquez, A. Ansiedad y depresión en primigestas adolescentes y añosas según la edad gestacional. Tesis doctoral, Universidad Central de Venezuela, Caracas. 1995

[39] Van Heyningen T.; Honikman S.; Myer L.; Onah M.N.; Field S.; Tomlinson M. Prevalence and predictors of anxiety disorders amongst low-income pregnant women in urban South Africa: a cross-sectional study. Arch Womens Ment Health. 2017, 20(6), 765–775. 10.1007/s00737-017-0768-z 28852868 PMC6086488

[40] Lardon E, St-Laurent A, Babineau V, Descarreaux M, Ruchat SM. Lumbopelvic pain, anxiety, physical activity and mode of conception: a prospective cohort study of pregnant women. BMJ Open. 2018;8(11):e022508 10.1136/bmjopen-2018-022508 30389759 PMC6224755

[41] Darwiche J, Milek A, Antonietti JP, Vial Y. Partner support during the prenatal testing period after assisted conception. Women Birth. 2019 4;32(2):e264–e271. 10.1016/j.wombi.2018.07.006 30100195

